# The History and Capabilities of Herbal Simples

**Published:** 1890-04-05

**Authors:** W. T. Fernie


### The History and Capabilities of Herbal Simples.

By W. T. Fernie, M.D.

*(Continued from page 355, Vol. VII.)*

### VII—WOOD BETONY.

Few, if any, herbal plants have been more praised for their curative virtues than the Wood Betony, Stachys Betonica, of the Labiatæ, or lipped order. By common people it is often called Bitny. The name Betonica is altered from the Celtic bentonic: ben=head, and tonic=good, in allusion to the usefulness of the plant against infirmities of the head. It is of common growth in shady woods and meadows, has aromatic leaves, and spikes of light purple flowers; formerly it was held in the very highest esteem as a healing simple. The Greeks loudly extolled its good qualities. Pliny in downright raptures styled it, "ante cunctas laudatissima." Antoninus Musa, chief physician to Cæsar Augustus, wrote a book entirely on the good properties of the Betony. An old Italian proverb ran thus:—"Vende la tunica, en compra la Betonia,"—sell your coat and buy Betony; whilst the modern Italians, when speaking of a superlatively good man, say, "He has as many virtues as the betony." Later on, Culpeper said, "This is a precious herb well worth keeping in your house;" and Gerard has told us: "Betony maketh a man to have a good appetite to his meate; and is to be commended against ache of the huckle-bone; it is good also for those who have ill heads upon a cold cause, and hath great virtue to heal the body being hurt within by bruising, or such like." Bartholinus relates that persons employed in gathering betony for medical purposes, have become partially intoxicated, so as then to commit various extravagancies. In an old standard work, "Medicina Britannica," 1666, the author writes:—"I have known the most obstinate headaches cured by daily breakfasting for a month or six weeks on a decoction of wood betony in new milk and strained." Likewise Dr. Meyrick has testified that inveterate headaches, after resisting every other remedy, have been cured by taking daily at breakfast a decoction made from the leaves and tops of the betony. For the same purpose the flowers and leaves have been dried and smoked as tobacco, and when powdered, they used to form an ingredient in Rowley's British Herb Snuff, which was at one time quite famous. Notwithstanding all this consensus of praise from writers, both ancient and modern, I cannot find that the betony possesses any special medicinal, or curative properties as shown by chemical analysis, or precise research. It seems only to afford simple fragrant aromatic principles, in common with most other plants of the Labiate order. Such aromatic vegetation is a feature of deserts, and of other hot soils all over the world. Scientists now suggest that the presence of essential oils in the leaves of these plants is for protecting them against the intense dry heat of a desert sun as effectively as if they were partly under shelter. Tyndall has shown the power exercised by a spray of perfume when diffused through a room to cool it, or, in other words, to exclude the passage of the heat rays. Therefore, whilst willing to pay all proper respect to the wood betony, I am inclined concerning it to side with Johnson, who says:—"In writing on plants some have given us counterfeit figures, as Matthiolus, or Dioscorides; and others counterfeit cures, which Crato avers were 'Potius fictæ, quam factæ,' "rather fictions than facts." Parkinson who enlarged Gerard's Herbal, found the root of the wood betony to be of much differing quality from the leaves and flowers, as being displeasing alike to the taste and stomach, whereas the leaves and flowers by their sweet and spicy taste are comfortable both in meate, and medicine." Anyhow, betony tea is a safe drink, and may still be found very beneficial against languid nervous headaches. It should be made by pouring half a pint of boiling water on a small handful of the leaves and tops, freshly gathered. A teacupful may be taken at once, and repeated in a quarter of an hour, if still needed. Both this plant and the water betony—so called from its similarity of leaf—bear the name of kernel-wort, from having tubers or kernels attached to the roots, and from being therefore supposed, according to the doctrine of signatures, to cure diseased kernels or scrofulous glands in the neck. In "the History of Brutes," [Franzius] we read concerning the stag, "when he is wounded with the dart, the only cure he hath is to eate some of the herbe called betony, which helpeth both to draw out the dart and to heal the wound."

